# Coexistence of bone and vascular disturbances in patients with endogenous glucocorticoid excess

**DOI:** 10.1016/j.bonr.2022.101610

**Published:** 2022-08-11

**Authors:** Chieko Yano, Maki Yokomoto-Umakoshi, Masamichi Fujita, Hironobu Umakoshi, Seiichi Yano, Norifusa Iwahashi, Shunsuke Katsuhara, Hiroki Kaneko, Masatoshi Ogata, Tazuru Fukumoto, Eriko Terada, Yayoi Matsuda, Ryuichi Sakamoto, Yoshihiro Ogawa

**Affiliations:** Department of Medicine and Bioregulatory Science, Graduate School of Medical Sciences, Kyushu University, Fukuoka, Japan

**Keywords:** Glucocorticoid, Bone, Vascular, Osteoporosis, Atherosclerosis, Endogenous Cushing's syndrome

## Abstract

**Purpose:**

Bone and vascular diseases are considered to share pathogenic mechanisms. Excess glucocorticoids, key regulators of cardiovascular and metabolic homeostasis, may promote both diseases simultaneously. We used endogenous Cushing's syndrome (CS) to investigate whether glucocorticoid excess underlies coexisting bone and vascular diseases.

**Methods:**

We included 194 patients with adrenal tumors (ATs): autonomous cortisol secretion (ACS, n = 97) and non-functional AT (n = 97). ACS was further classified into overt CS (n = 17) and subclinical CS (SCS, n = 80). Arterial stiffness was defined as a brachial-ankle pulse wave velocity (baPWV) ≥ 1800 cm/s.

**Results:**

Patients with ACS had higher coexistence rates of vertebral fracture and arterial stiffness (23 % vs. 2 %; p < 0.001) and vertebral fracture and abdominal aortic calcification (22 % vs. 1 %; p < 0.001) than those with non-functional AT. In patients with ACS, baPWV was negatively correlated with trabecular bone score (TBS, r = −0.33; p = 0.002), but not with bone mineral density, and vertebral fracture was associated with arterial stiffness in the logistic regression analysis. In the multivariate analysis of variance, the degree of cortisol excess (defined as CS, SCS, and non-functional AT) determined the correlation between TBS and baPWV (partial η^2^ = 0.07; p < 0.001). In the analysis of covariance, patients with coexisting vertebral fracture and arterial stiffness had higher levels of serum cortisol after the 1-mg dexamethasone suppression test than those without.

**Conclusion:**

In endogenous glucocorticoid excess, bone and vascular diseases frequently coexisted, and deteriorated bone quality, not bone loss, was related to arterial stiffness. Thus, glucocorticoid excess may perturb the bone-vascular axis.

## Highlights

• Endogenous Cushing's syndrome (CS) often coexisted bone and vascular diseases.
• In CS, deteriorated bone quality, not bone loss was related to arterial stiffness.
• Glucocorticoid excess may perturb the bone-vascular axis.

## Graphical abstract

**Figure:**
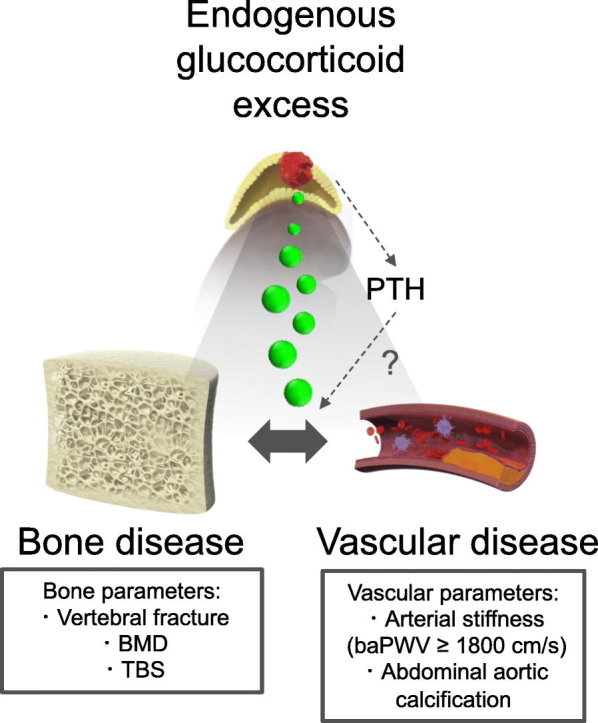

## 1 Introduction

Long-term exposure to glucocorticoids due to endogenous glucocorticoid excess in Cushing's syndrome (CS) or exogenous glucocorticoid administration is associated with various cardiometabolic complications (Newell-Price et al., 2006). Glucocorticoid-induced osteoporosis results in decreased bone strength and an increased risk of fracture, which greatly reduces the quality of life (Hermus et al., 1995; Tauchmanovà et al., 2006; Chiodini et al., 2009). Further, patients with endogenous glucocorticoid excess have a higher risk of death compared to the general population, mainly due to cardiovascular diseases (van Haalen et al., 2015), and often develop atherosclerosis (Faggiano et al., 2003; Neary et al., 2013).

Bone and vascular diseases often coexist, both being common age-related diseases (Tankó et al., 2005; Lewis et al., 2019; Raisi-Estabragh et al., 2021). They share risk factors and pathogenic mechanisms, which are known as the “bone-vascular axis” (Thompson and Towler, 2012). Dysmetabolic states, such as diabetes mellitus and hyperlipidemia, perturb the bone-vascular axis, thus allowing both diseases to develop simultaneously (Thompson and Towler, 2012; Wagenknecht et al., 2016). Previous experimental studies suggest that excess glucocorticoids, key regulators of cardiovascular and metabolic homeostasis, also perturb the bone-vascular axis. Particularly, exogenous glucocorticoid administrations cause vascular calcification by promoting osteogenic differentiation of vascular cells (Kirton et al., 2006; Mori et al., 1999). However, whether glucocorticoid excess promotes the coexistence of bone and vascular diseases in humans remains unclear.

Since endogenous CS occurs rarely, much of our knowledge about glucocorticoid-induced osteoporosis and atherosclerosis have been from studies of patients treated with exogenous glucocorticoids (Tóth and Grossman, 2013). These studies are limited by the fact that patients treated with glucocorticoids usually have primary disease(s) that may affect bone and vascular conditions. In this regard, endogenous CS would provide a better understanding of the direct effect of glucocorticoid excess on bone and vascular pathologies. The aim of the present study was to investigate how endogenous glucocorticoid excess is involved in the coexistence of bone and vascular diseases.

## 2 Materials and methods

### 2.1 Study design

This was a retrospective cross-sectional study conducted at the Kyushu University Hospital, a referral center in Japan. The study protocol was approved by the Institutional Ethics Committee (No. 21025-00). The study was performed in accordance with the guidelines for clinical studies published by the Ministry of Health and Labour, Japan. Informed consent was obtained from all patients upon hospital admission.

### 2.2 Patients

**Fig. 1:**
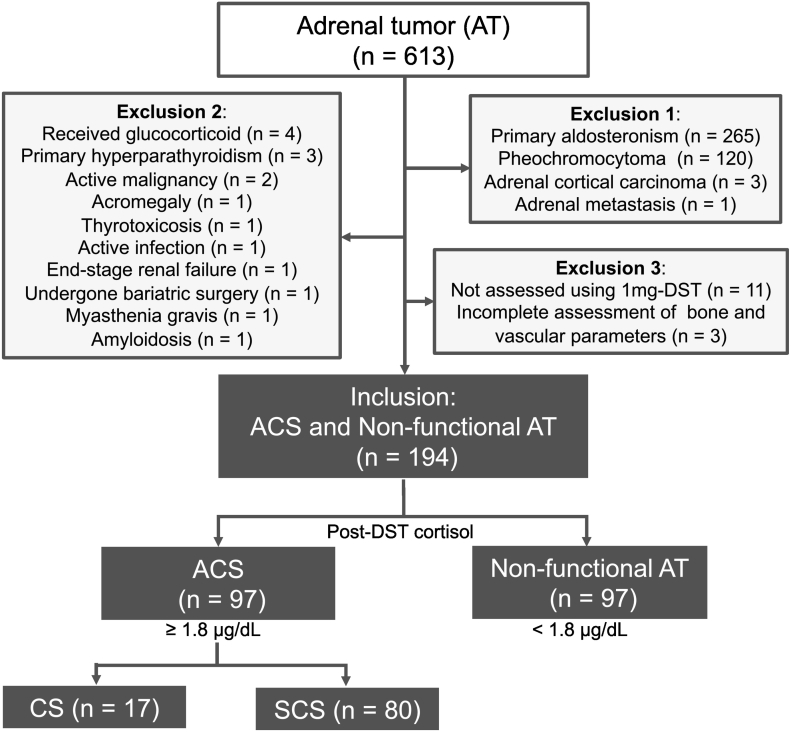
Study design. The flowchart shows the process of patient selection in the study. Abbreviations: ACS, autonomous cortisol secretion; AT, adrenal tumor; DST, dexamethasone suppression test; CS, overt Cushing's syndrome; SCS, subclinical Cushing's syndrome; BMD, bone mineral density; TBS, trabecular bone score; baPWV, brachial-ankle pulse wave velocity, PTH, parathyroid hormone.

We enrolled 613 consecutive patients with adrenal tumors (ATs), who were admitted to our endocrine unit between January 2013 and September 2021 (Fig. 1). Patients with primary aldosteronism (n = 265), pheochromocytoma (n = 120), adrenal cortical carcinoma (n = 3), or adrenal metastasis (n = 1) were excluded. Patients with conditions that are known to affect bone or vascular metabolism (e.g., glucocorticoid treatment, primary hyperparathyroidism, and active malignancies) were excluded (n = 16). The patients who were not assessed using the 1-mg dexamethasone suppression test (1 mg-DST; n = 11) or with those with an incomplete assessment of bone and vascular parameters (n = 3) were excluded. Finally, we included 194 patients who were examined for both bone and vascular parameters, of whom 97 had autonomous cortisol secretion (ACS) and 97 had non-functional AT as a control. None of patients included in this study were treated with antiresorptive drugs (bisphosphonates, denosumab) and osteoanabolic drug (teriparatide). None of the patients in this study were also supplemented with calcium or vitamin D as medical treatment, but we were unable to ascertain whether patients were taking them personally. At least one of the following bone parameters was examined: vertebral fracture, bone mineral densities (BMDs) at the lumbar spine and femoral neck, and trabecular bone score (TBS). Vascular parameters were examined for brachial-ankle pulse wave velocity (baPWV), abdominal aortic calcification, or both, which are the standard measures for assessing atherosclerosis (Townsend et al., 2015; Szulc, 2016). Patients with ACS and non-functional AT were diagnosed based on a serum cortisol level after 1 mg-DST (hereafter referred to as “post-DST cortisol”) ≥ 1.8 μg/dL (50 nmol/L) and <1.8 μg/dL, respectively. Patients with ACS (n = 97) were divided into those with overt CS (CS; n = 17) and those with subclinical CS (SCS; n = 80). CS was diagnosed based on clinical findings in the Clinical Practice Guidelines of the Endocrine Society (Nieman et al., 2008). SCS was diagnosed when a post-DST cortisol level ≥ 1.8 μg/dL (50 nmol/L), but the patient had no typical symptoms (Fassnacht et al., 2016).

### 2.3 Data collection for baseline characteristics

Information regarding alcohol intake (≤ or >3 units/day), smoking status (past or current), and menopause status was obtained using an interviewer-assisted questionnaire. Diabetes mellitus was diagnosed when the patient had glycosylated hemoglobin ≥6.5 %, a fasting glucose level ≥ 126 mg/dL, or a history of antidiabetic medication. Hypertension was diagnosed when the patient had a history of antihypertensive medication or a 24 h blood pressure ≥ 140/90 mmHg. Hyperlipidemia was diagnosed when the patient had a serum low-density lipoprotein-cholesterol level ≥ 140 mg/dL or a history of lipid-lowering medication.

### 2.4 Evaluation of bone parameters

Lateral radiographs of the thoracic and lumbar spines were examined to diagnose vertebral fractures, a hallmark of osteoporosis (Kanis, 2002), using the Genant semi-quantitative visual assessment (Genant et al., 1993), as described previously (Yokomoto-Umakoshi et al., 2020a). “Severe vertebral fracture” was diagnosed when the patient had multiple vertebral fractures or a grade-3 vertebral fracture (Soen et al., 2013). Two trained investigators, who were blinded to each other's assessment, obtained the diagnoses, with an interrater reliability of 0.80. A third investigator resolved the disagreements. None of the patients had a history of severe trauma. BMDs at the lumbar spine (L1-L4) and femoral neck were obtained by dual-energy X-ray absorptiometry using the Discovery A system (Hologic, Waltham, MA, US), with coefficients of variation of 0.93 % and 1.74 %, respectively. TBS was retrospectively analyzed in a blinded manner from BMD at the lumbar spine using TBS iNsight software, version 3.0.3.0 (Medimaps, France), with a coefficient of variation of 1.12 %. All patients evaluated for TBS were older than 20 years and had a body mass index (BMI) ≤ 35 kg/m^2^.

### 2.5 Evaluation of vascular parameters

The baPWV was measured in the supine position after 15 min of rest using an automatic waveform analyzer (Omron Colin Co., Ltd., Komaki, Japan). Arterial stiffness was diagnosed when the patient had a baPWV ≥1800 cm/s (Munakata, 2014). Abdominal aortic calcification was diagnosed using the semi-quantitative method by Kauppila et al. (Kauppila et al., 1997) and lateral radiographs of the lumbar spine as described previously (Yokomoto-Umakoshi et al., 2020b). Abdominal aortic calcification was diagnosed when the patient scored ≥3 (Bagger et al., 2006). Two investigators blinded to each other's assessment obtained the diagnoses, with an interrater reliability of 0.93. A third investigator resolved the disagreements.

### 2.6 Biochemical measurements and assay methods

We collected basal serum cortisol at 0800 h (reference range, 6.2–18.0 μg/dL), midnight serum cortisol at 2300 h, morning plasma adrenocorticotropic hormone (ACTH), 24 h urinary free cortisol, and post-DST cortisol. For post-DST cortisol, 1 mg of dexamethasone had been administered at 2300 h the day before the collection. Their reference ranges and assay methods have been described previously (Yokomoto-Umakoshi et al., 2020c). The estimated glomerular filtration rate (eGFR), serum calcium level, intact-parathyroid hormone (PTH) level, urinary calcium-to-creatinine ratio, tartrate-resistant acid phosphatase-5b (TRACP-5b) level, and bone-alkaline phosphatase (BAP) level were measured, as described previously (Yokomoto-Umakoshi et al., 2020c). Dehydroepiandrosterone sulfate (DHEAS) and 25-hydroxyvitamin D levels were measured using electrochemiluminescence and chemiluminescent enzyme immunoassays, respectively.

### 2.7 Statistical analysis

The clinical characteristics between patients with ACS and those with non-functional AT were compared using the Mann-Whitney U or Fischer's exact test, as appropriate. In patients with ACS, the correlation between bone and vascular parameters was examined using Spearman's test and after adjusting for possible confounders, we examined the association using logistic regression analysis. Further, clinical characteristics among patients with CS, SCS, and non-functional AT were compared using the Fischer's exact or Kruskal-Wallis test, as appropriate, with post-hoc Bonferroni correction. Multivariate analysis of variance (MANOVA) was performed to evaluate whether cortisol excess was a determinant of the correlated bone and vascular parameters. The analysis of covariance (ANCOVA) was performed to evaluate the differences in post-DST cortisol between patients with and without coexisting bone and vascular diseases. Possible confounders included age, gender, BMI, alcohol intake, smoking status, and the presence of diabetes mellitus, hypertension, and hyperlipidemia, all of which affected bone and vascular diseases (Ensrud and Crandall, 2017; Herrington et al., 2016). Continuous variables with skewed distributions were log-transformed, as appropriate. All tests were two-tailed, and p < 0.05 was set as statistical significance. Statistical analyses were performed using R software (version 4.0.3) (R Core Team, 2013).

## 3 Results

### 3.1 Comparison of clinical characteristics between patients with ACS and those with non-functional AT

Clinical characteristics between patients with ACS (n = 97) and those with non-functional AT (n = 97) were compared (Table 1). Patients with ACS had higher levels of basal serum cortisol, midnight serum cortisol, 24 h urinary free cortisol, and post-DST cortisol. Further, they had higher frequencies of diabetes mellitus, hypertension, and hyperlipidemia and lower levels of ACTH and DHEAS. Regarding bone parameters, patients with ACS had a higher frequency of vertebral fracture, lower BMD Z score at the lumbar spine, femoral neck and TBS; and a higher intact-PTH level and urinary calcium-to-creatinine ratio than those with non-functional AT. Regarding vascular parameters, patients with ACS had higher frequencies of arterial stiffness and abdominal aortic calcification than those with non-functional AT. Furthermore, patients with ACS had higher rates of coexistence of vertebral fracture and arterial stiffness (23 %, 18/76 vs. 2 %, 2/83; p < 0.001) and vertebral fracture and abdominal aortic calcification (22 %, 17/77 vs. 1 %, 1/84; p < 0.001) than those with non-functional AT. There was no significant difference in age, gender, menopausal status, BMI, alcohol intake, smoking status, eGFR, serum calcium, 25-hydroxyvitamin D, TRACP-5b, or BAP level between the two groups.

**Table 1:** Comparison of clinical characteristics between patients with ACS and those with non-functional AT.

Variables		ACS (n = 97)	Non-functional AT (n = 97)	p-Value
Baseline parameters	Age, y	62.0 [45.0, 69.0]	58.0 [51.0, 67.0]	0.840
	Gender, female, %	61 % (60/97)	53 % (52/97)	0.309
	Postmenopausal female, %	58 % (35/60)	73 % (38/52)	0.116
	BMI, kg/m^2^	24.1 [21.3, 26.9]	24.1 [21.8, 27.1]	0.465
	Alcohol intake, %	48 % (46/95)	45 % (44/97)	0.772
	Smoking status, %	53 % (51/95)	44 % (43/97)	0.248
	Basal serum cortisol, μg/dL	14.6 [10.5, 18.9]	12.7 [9.1, 15.9]	0.024⁎
	ACTH, pg/mL	6.8 [1.5, 15.7]	24.0 [16.6, 37.1]	<0.001⁎
	Midnight serum cortisol, μg/dL	6.0 [4.5, 11.5]	2.9 [2.0, 4.2]	<0.001⁎
	Post-DST cortisol, μg/dL	4.0 [2.5, 12.3]	1.1 [0.8, 1.3]	<0.001⁎
	24 h urinary free cortisol, μg/day	59.4 [36.7, 90.3]	43.7 [31.8, 59.8]	0.003⁎
	DHEAS, μg/dL	38.0 [15.7, 65.5]	92.0 [61.5, 142.5]	<0.001⁎
	eGFR, ml/min/1.73m^2^	81.0 [65.4, 94.9]	81.0 [71.0, 93.0]	0.998
	Diabetes mellitus, %	44 % (43/97)	22 % (22/97)	0.002⁎
	Hypertension, %	71 % (69/96)	52 % (50/96)	0.007⁎
	Hyperlipidemia, %	56 % (55/97)	36 % (35/97)	0.006⁎
Bone parameters	Vertebral fracture, %	49 % (38/77)	8 % (7/84)	<0.001⁎
	Severe vertebral fracture, %	16 % (13/77)	2 % (2/84)	0.002⁎
	BMD at lumbar spine, g/cm^2^	0.87 [0.74, 1.00] (n = 89)	0.88 [0.80, 1.06] (n = 87)	0.065
	BMD at lumbar spine Z score	−0.2 [−0.9, 0.7] (n = 89)	0.1 [−0.6, 0.9] (n = 87)	0.043⁎
	BMD at femoral neck, g/cm^2^	0.62 [0.53, 0.72] (n = 94)	0.65 [0.60, 0.76] (n = 87)	0.010⁎
	BMD at femoral neck Z score	−0.6 [−1.2, 0.3] (n = 94)	−0.3 [−0.8, 0.4] (n = 87)	0.025⁎
	TBS	1.34 [1.28, 1.39] (n = 82)	1.37 [1.31, 1.42] (n = 82)	0.026⁎
	Serum calcium, mg/dL	9.3 [9.1, 9.5]	9.3 [9.1, 9.5]	0.312
	Intact-PTH, pg/mL	53.8 [38.3, 69.2] (n = 91)	43.8 [34.5, 54.7] (n = 94)	0.006⁎
	Urinary calcium-to-creatinine ratio	0.17 [0.11, 0.22] (n = 69)	0.13 [0.08, 0.20] (n = 75)	0.049⁎
	25-hydroxyvitamin D, ng/mL	13.7 [9.7, 17.5] (n = 44)	14.3 [10.5, 18.5] (n = 68)	0.331
	TRACP-5b, mU/dL	358 [245, 454] (n = 87)	323 [251, 433] (n = 95)	0.514
	BAP, μg/L	12.3 [9.8, 15.4] (n = 86)	12.5 [9.9, 15.9] (n = 94)	0.734
Vascular parameters	baPWV, cm/s	1675 [1451, 1891] (n = 96)	1541 [1365, 1707] (n = 95)	0.007⁎
	Arterial stiffness, %	37 % (36/95)	21 % (20/95)	0.017⁎
	Abdominal aortic calcification, %	32 % (25/77)	15 % (13/84)	0.015⁎
Coexistence rates of bone and vascular diseases	Vertebral fracture and arterial stiffness, %	23 % (18/76)	2 % (2/83)	<0.001⁎
	Vertebral fracture and abdominal aortic calcification, %	22 % (17/77)	1 % (1/84)	<0.001⁎

Data are expressed as median [interquartile range] or percentage (number of patients). Severe vertebral fracture was defined as multiple vertebral fractures or a grade 3 vertebral fracture. Arterial stiffness was defined as a baPWV ≥1800 cm/s.

Abbreviations: ACS, autonomous cortisol secretion; AT, adrenal tumor; BMI, body mass index; ACTH, adrenocorticotropic hormone; DST, dexamethasone suppression test; DHEAS, dehydroepiandrosterone sulfate; eGFR, estimated glomerular filtration rate; PTH, parathyroid hormone; TRACP-5b, tartrate-resistant acid phosphatase-5b; BAP, bone-alkaline phosphatase; BMD, bone mineral density; TBS, trabecular bone score; baPWV, brachial-ankle pulse wave velocity.

⁎ p < 0.05 was considered significant.

### 3.2 Correlation between bone and vascular parameters in patients with ACS

**Fig. 2:**
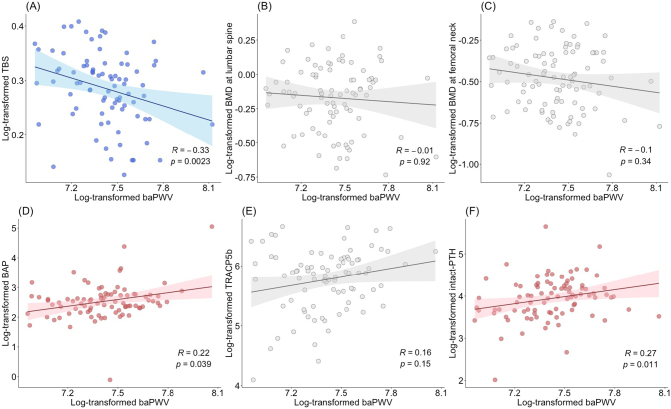
Correlation between bone and vascular parameters in patients with ACS. (A) TBS was negatively correlated with baPWV (n = 82). (B, C) There were no correlations between BMD at lumbar spine and femoral neck and baPWV (n = 88 and n = 93, respectively) (D, F) BAP and intact-PTH levels were positively correlated with baPWV (n = 85 and n = 90, respectively). (E) There was no correlation between TRACP-5b levels and baPWV (n = 86). In these analyses, the TBS, BMD, BAP level, TRACP5b level, intact-PTH level, and baPWV were log-transformed. *, p < 0.05 was considered significant. Abbreviations: ACS, autonomous cortisol secretion; baPWV, brachial-ankle pulse wave velocity; TBS, trabecular bone score; BMD, bone mineral density; BAP, bone-alkaline phosphatase, TRACP-5b, tartrate-resistant acid phosphatase-5b; PTH, parathyroid hormone.

We examined the correlation between bone parameters and baPWV in patients with ACS. In these analyses, the TBS, BMD, baPWV, and the levels of BAP, TRACP5b, and intact-PTH were log-transformed. TBS was negatively correlated with baPWV (n = 82, r = −0.33; p = 0.002; Fig. 2A). In contrast, BMD at the lumbar spine (n = 88) or femoral neck (n = 93) and baPWV showed no correlation (Fig. 2B and C). BAP and intact-PTH levels were positively correlated with baPWV (n = 85, r = 0.22; p = 0.039 and n = 90, r = 0.27; p = 0.011, respectively; Fig. 2D and F). Further, TRACP-5b level and baPWV showed no correlation (n = 86; Fig. 2E).

### 3.3 Association between bone and vascular parameters in the patients with ACS after adjusting for possible confounders

We examined the association between vertebral fractures and arterial stiffness in patients with ACS (Table 2). In these analyses, age and BMI were log-transformed. Vertebral fracture was associated with arterial stiffness (n = 76; odds ratio [OR] = 2.74; 95 % confidence interval [CI]: 1.06–7.42; p = 0.048). The association remained significant even after adjusting for age, gender, BMI, alcohol intake, smoking status, and the presence of diabetes mellitus in model 1 (OR = 3.29; 95 % CI: 1.07–10.9; p = 0.041), but attenuated in models 2 and 3 (diabetes mellitus of model 1 replaced by hypertension and hyperlipidemia, respectively; p = 0.070 and p = 0.076, respectively). Severe vertebral fracture was associated with arterial stiffness (n = 76; p = 0.026), and the association remained significant even after the adjustment in models 1–3 (p < 0.05). In addition, vertebral fracture was associated with abdominal aortic calcification (n = 77; p = 0.026; Supplementary Table 1). The association remained significant even after the adjustment in model 1 (p = 0.048), but was attenuated in models 2 and 3 (p = 0.077 and p = 0.063, respectively).

**Table 2:** Association between bone and vascular parameters in patients with ACS after adjusting for possible confounders.

Variables	OR	95%CI	p-Value
Association between vertebral fracture and arterial stiffness (n = 76)			
Crude	2.74	1.06–7.42	0.040⁎
Model 1	3.29	1.07–10.9	0.041⁎
Model 2	2.94	0.93–9.97	0.070
Model 3	2.70	0.91–8.48	0.076

Association between severe vertebral fracture and arterial stiffness (n = 76)			
Crude	4.40	1.23–18.1	0.026⁎
Model 1	9.36	1.90–63.7	0.010⁎
Model 2	6.01	1.19–41.2	0.041⁎
Model 3	5.33	1.26–28.7	0.031⁎

Arterial stiffness was defined as a baPWV ≥1800 cm/s. Severe vertebral fracture was defined as multiple vertebral fractures or a grade 3 vertebral fracture. In these analyses, age, gender, BMI, alcohol intake, smoking status, and the presence of diabetes mellitus were adjusted in model 1, and diabetes mellitus of model 1 was replaced by hypertension and hyperlipidemia in models 2 and 3, respectively. Age and BMI were log-transformed.

Abbreviations: ACS, autonomous cortisol secretion; baPWV, brachial-ankle pulse wave velocity; OR, odds ratio; CI, confidence interval; BMI, body mass index.

⁎ p < 0.05 was considered significant.

### 3.4 Comparison of clinical characteristics among patients with CS, SCS, and non-functional AT

We compared the clinical characteristics among the three groups of patients with CS (n = 17), SCS (n = 80), and non-functional AT (n = 97) (Supplementary Table 2). Patients with CS were younger than those with SCS and non-functional AT. Other baseline parameters, such as gender, BMI, alcohol intake, or smoking status, showed no significant difference. The rate of coexistence of vertebral fracture with arterial stiffness and abdominal aortic calcification showed an association among the three groups (p < 0.001 for both).

### 3.5 Cortisol excess as a determinant of the correlation between bone and vascular parameters

The MANOVA was performed to evaluate whether an excess of cortisol determined the correlation between bone and vascular parameters (Table 3). In these analyses, the degree of cortisol excess was the independent variable (defined as CS, SCS, and non-functional AT), and baPWV and bone parameters (TBS, BAP level, and intact- PTH level) were the dependent variables. TBS, BAP level, intact-PTH level, baPWV, age, and BMI were log-transformed. The degree of cortisol excess determined the correlation between TBS and baPWV after adjusting for age, gender, BMI, alcohol intake, smoking status, and the presence of diabetes mellitus in model 1 (n = 164; Pillai's trace = 0.14, F (4, 308), 5.86; p < 0.001; partial η^2^ = 0.07). The results were similar when diabetes mellitus in model 1 was replaced by hypertension and hyperlipidemia in models 2 and 3, respectively. The degree of cortisol excess determined the correlation between BAP level and baPWV (n = 177; p < 0.001; partial η^2^ = 0.05), and between intact-PTH level and baPWV (n = 184; p = 0.006; partial η^2^ = 0.04), after the adjustment in model 1. Similar results were obtained in models 2 and 3.

**Table 3:** Cortisol excess as a determinant of the correlation between bone and vascular parameters.

Dependent variables	N	Pillai's trace	F	Num df	Den df	p-Value	Partial η^2^
TBS and baPWV							
Model 1	164	0.14	5.86	4	308	<0.001⁎	0.07
Model 2		0.14	5.71			<0.001⁎	0.07
Model 3		0.14	5.86			<0.001⁎	0.07

BAP and baPWV							
Model 1	177	0.10	4.77	4	348	<0.001⁎	0.05
Model 2		0.10	4.98			<0.001⁎	0.05
Model 3		0.10	4.79			<0.001⁎	0.05

Intact-PTH and baPWV							
Model 1	184	0.08	3.67	4	334	0.006⁎	0.04
Model 2		0.08	3.82			0.004⁎	0.04
Model 3		0.08	3.67			0.006⁎	0.04

In these analyses, the degree of cortisol excess (defined as CS, SCS, and non-functional AT) was the independent variable. Age, gender, BMI, alcohol intake, smoking status, and the presence of diabetes mellitus were adjusted in model 1, and diabetes mellitus of model 1 was replaced by hypertension and hyperlipidemia in models 2 and 3, respectively. The TBS, BAP level, intact-PTH level, baPWV, age, and BMI were log-transformed.

Abbreviations: MANOVA, multivariate analysis of variance; Num df, numerator degrees of freedom; Den df, denominator degrees of freedom; CS, overt Cushing's syndrome; SCS, subclinical Cushing's syndrome; AT, adrenal tumor; baPWV, brachial-ankle pulse wave velocity; TBS, trabecular bone score; BAP, bone-alkaline phosphatase; PTH, parathyroid hormone; BMI, body mass index.

⁎ p < 0.05 was considered significant.

### 3.6 Differences in post-DST cortisol between patients with and without coexisting bone and subclinical vascular diseases

**Fig. 3:**
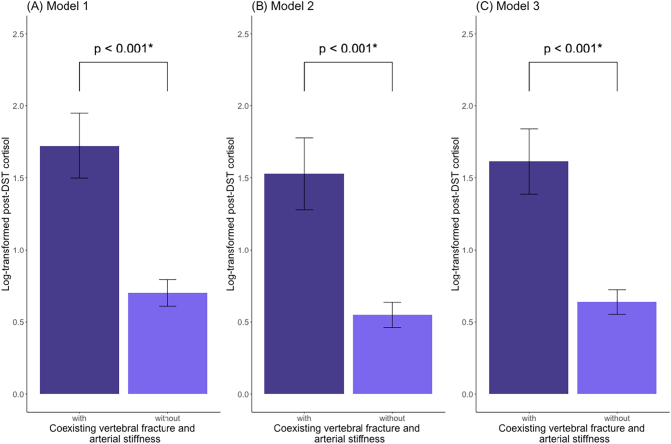
Differences in post-DST cortisol between patients with and without coexisting bone and subclinical vascular diseases. (A) Patients with coexisting vertebral fracture and arterial stiffness (n = 20) had higher levels of post-DST cortisol than those without (n = 139) after adjusting for age, gender, BMI, alcohol intake, smoking status, and the presence of diabetes mellitus in model 1. (B, C) The results were similar when diabetes mellitus in model 1 was replaced by hypertension and hyperlipidemia in models 2 and 3, respectively. In these analyses, data are expressed as the LSM with 95 % CI from ANCOVA after adjustment in models 1–3. The Post-DST cortisol, age, and BMI were log-transformed. Arterial stiffness was defined as baPWV ≥1800 cm/s. *, p < 0.05 was considered significant. Abbreviations: CS, overt Cushing's syndrome; SCS, subclinical Cushing's syndrome; AT, adrenal tumor; DST, dexamethasone suppression test; baPWV, brachial-ankle pulse wave velocity; ANCOVA, analysis of covariance; LSM, least-square mean; CI, confidence intervals; BMI, body mass index.

The ANCOVA was performed to compare post-DST cortisol between patients with and without coexisting vertebral fracture and arterial stiffness. In these analyses, the post-DST cortisol, age, and BMI were log-transformed. Patients with both vertebral fracture and arterial stiffness (n = 20) had higher levels of post-DST cortisol than those without (n = 139) after adjusting for age, gender, BMI, alcohol intake, smoking status, and the presence of diabetes mellitus in model 1 (least-squares mean [95 % CI]: 1.72 [1.49–1.94] vs. 0.70 [0.60–0.79]; p < 0.001; Fig. 3A). The results were similar when diabetes mellitus in model 1 was replaced by hypertension and hyperlipidemia in models 2 and 3, respectively (Fig. 3B and C). In models 1–3, patients with coexisting vertebral fracture and abdominal aortic calcification had higher levels of post-DST cortisol than those without this coexistence (n = 18 and 143, respectively) (Supplementary Fig. 1A-1C).

In addition, we adjusted simultaneously for the 3 variables of the presence of diabetes mellitus, hypertension, and hyperlipidemia, with results similar to those obtained when adjusted individually. Patients with both vertebral fracture and arterial stiffness had higher levels of post-DST cortisol than those without after adjusting for age, gender, BMI, alcohol intake, smoking status, the presence of diabetes mellitus, hypertension, and hyperlipidemia (least-squares mean [95 % CI]: 1.48 [1.24–1.73] vs. 0.63 [0.54–0.72]; p = 0.005) (Supplementary Fig. 2A). Similar results were obtained when comparing patients with both vertebral fractures and abdominal aortic calcification with those without (least-squares mean [95 % CI]: 1.44 [1.19–1.69] vs. 0.67 [0.58–0.76]; p = 0.001) (Supplementary Fig. 2B).

## 4 Discussion

This study demonstrated that patients with endogenous glucocorticoid excess frequently developed coexisting vertebral fracture and arterial stiffness, or vertebral fracture and abdominal aortic calcification. Further, endogenous glucocorticoid excess was associated with each of bone and vascular disease, which was consistent with previous reports (Hermus et al., 1995; Tauchmanovà et al., 2006; Chiodini et al., 2009; van Haalen et al., 2015; Faggiano et al., 2003; Neary et al., 2013). Patients with both vertebral fractures and arterial stiffness showed higher levels of post-DST cortisol than those without these conditions, irrespective of the dysmetabolic state. Previous clinical studies addressed the association of endogenous glucocorticoid excess with bone or vascular disease; however, both bone and vascular diseases were not investigated together. These observations suggested that excess endogenous cortisol leads to the progression of bone and subclinical vascular diseases simultaneously. Considering that exogenous glucocorticoid excess can be confounded by the primary disease, our findings obtained in patients with endogenous CS support the notion that glucocorticoid excess perturbs the bone-vascular axis.

In vitro evidence suggests that a possible mechanism through which glucocorticoid excess perturbs the bone-vascular axis is that glucocorticoid administration increases alkaline phosphatase activity and calcification (i.e., osteogenic differentiation) in vascular smooth muscle cells and vascular pericytes (Kirton et al., 2006; Mori et al., 1999). In this study, we found that endogenous glucocorticoid excess determined the positive correlation between the BAP level and baPWV, suggesting that the BAP level reflects osteogenic differentiation of vascular cells and the development of vascular disease. Furthermore, we need to consider not only the direct effects of glucocorticoid excess on bone and vascular diseases, but also the indirect effects via PTH. Glucocorticoid excess is known to cause secondary hyperparathyroidism (Hardy et al., 2018), as patients with ACS had elevated levels of urinary calcium excretion and serum intact-PTH in this study. PTH is an important regulator of bone and vascular disease; continuous exposure to PTH promotes bone remodeling, resulting in skeletal catabolism (Silva and Bilezikian, 2015). It also promotes vascular calcification and remodeling (Bernardi et al., 2021). In this study, glucocorticoid excess determined a positive correlation of the intact-PTH level with baPWV, suggesting that the effect of glucocorticoid excess on the bon-vascular axis is partially mediated by PTH action.

In patients with endogenous cortisol excess, TBS and baPWV showed a negative correlation, but BMD and baPWV showed no correlation, suggesting that deteriorated bone quality, and not decreased bone mass, was related to arterial stiffness. Glucocorticoid excess could have a greater influence on the bone microarchitecture than that on areal BMD (Dalle Carbonare et al., 2001). Further, TBS is an independent predictor of fractures in glucocorticoid-induced osteoporosis (Florez et al., 2020). Thus, our results may be explained in part by the notion that bone quality, and not bone mass, underlies the pathology of glucocorticoid-induced osteoporosis. Further studies are required to address whether or not oxidative stress, a major determinant of bone deterioration and atherosclerosis, mediates coexisting bone and vascular diseases in patients with endogenous glucocorticoid excess (Saito et al., 2014; Kattoor et al., 2017).

This study has clinical implications for the management of patients with excess glucocorticoids. Particularly, patients with arterial stiffness may have deteriorated bone quality, even without decreased bone mass. For patients with endogenous glucocorticoid excess in this study, arterial stiffness was an independent risk factor for severe vertebral fractures, which is a risk factor for future fractures (Kanis, 2002). For patients with glucocorticoid excess with arterial stiffness, we suggest the lateral radiographs of the thoracic and lumbar spines to confirm vertebral fracture even when they have normal BMD.

In addition to pathological glucocorticoid excess, mild glucocorticoid excess due to aging and chronic stress may be involved in the development of age-related diseases (van den Beld et al., 2018). Previous experimental studies have suggested that mild glucocorticoid excess with aging accelerates bone and vascular diseases simultaneously (Manolagas, 2010). Considering the increasing unmet clinical needs, such as therapeutic strategies that target common molecular pathways in age-related diseases (Thompson and Towler, 2012; Khosla et al., 2018), factors contributing to coexisting bone and vascular diseases should be investigated. This study provides clues to understand the role of glucocorticoid excess as a critical mediator of the bone-vascular axis.

When extrapolating our results to Cushing's disease by pituitary adenomas, it is necessary to consider that the pattern of adrenal steroid secretion is different from that of adrenal CS. ACTH-dependent Cushing's disease promotes steroid synthesis in the zona reticularis as well as in the zona fasciculata of adrenal cortex, and serum DHEAS levels are generally elevated; in contrast, they are often low in adrenal CS. DHEAS is a prohormone for sex hormone synthesis and has protective effects on bone and vascular diseases (Yokomoto-Umakoshi et al., 2021; Zhao et al., 2020). In fact, it has been reported that bone loss is milder in Cushing's disease than in adrenal CS (Minetto et al., 2004), and thus Cushing's disease and adrenal CS may have different coexistence rates of bone and vascular disease.

## 5 Limitations

This study has several limitations. First, it was a single center and retrospective study. Second, the sample size was small. In the logistic regression analysis, there was a tendency for an association between vertebral fracture and arterial stiffness, even after adjusting for possible confounders including the presence of hypertension or hyperlipidemia in patients with ACS, with no statistical significance. This may be partly because of the small sample size. Therefore, a multicenter prospective study should be conducted to validate our findings. Second, future mechanistic studies are needed to investigate the direct effects of glucocorticoid excess on the bone-vascular axis, independent of PTH action. Third, none of the patients in this study were also supplemented with calcium or vitamin D as medical treatment, but we were unable to ascertain whether patients were taking them personally. Fourth, this study was not able to evaluate the intima-media thickness or the coronary calcification score on CT. In addition, baPWV was examined as a part of routine medical care, and thus the reproducibility of the baPWV measurement could not be confirmed. The coefficients of variation of biochemical measurements such as intact-PTH, TRACP-5b, BAP, and 25-hydroxyvitamin D could not be evaluated. We could not even evaluation sclerostin. Osteocyte-derived protein sclerostin is a negative regulator of the Wnt/β-catenin signaling and is reported to prevent vascular calcification (De Maré et al., 2022). Glucocorticoid excess is associated with decreased circulating sclerostin levels in humans, suggesting that this may be due to decreased osteocyte number or function (van Lierop et al., 2012). Thus, sclerostin may be involved in the effects of glucocorticoid excess on bone-vascular axis. Furthermore, due to the small sample size of this study, it was not possible to simultaneously adjusting for the presence of diabetes, hypertension, and hyperlipidemia in the logistic regression analysis to examine the association between vertebral fractures and arterial stiffness. Finally, the selection of control patients could be a limitation, since a recent study reported that patients with non-functional AT are at an increased risk of cardiometabolic diseases (Lopez et al., 2016); hence, the control patients with non-functional AT may have a higher risk of bone and vascular diseases than the general population, which may underestimate the risks in patients with ACS.

## 6 Conclusion

In endogenous glucocorticoid excess, bone and vascular diseases frequently coexisted, and deteriorated bone quality, not bone loss, was related to arterial stiffness. This study may contribute to the appropriate management of such patients and provide new insights into our understanding of the bone-vascular axis.

## Data availability

Data supporting the findings presented in this study are available from the corresponding authors upon reasonable request.

## Funding

This work was supported in part by the grants from “JSPS KAKENHI” (JP20K16525 and 21J40043 to M. Y-U., 22H04993 to Y.O., and 22K08627 to H.U.), “The JSBMR Rising Stars Grant” (to M. Y-U.), “JAPAN Osteoporosis Foundation” (to M. Y-U.), “10.13039/100007449Takeda Science Foundation” (to H.U.), “10.13039/501100007206Kaibara Morikazu Medical Science Promotion Foundation” (to H.U. and M. Y-U.), “10.13039/100008695Japan Foundation for Applied Enzymology” (to H.U. and M. Y-U.), “10.13039/100008732The Uehara Memorial Foundation” (to H.U.), and “10.13039/501100004298Secom Science and Technology Foundation” (to Y.O.).

## CRediT authorship contribution statement

Conceptualization; CY, MY-U, YO, Data curation; CY, RS, YM, SK, NI, Formal analysis; CY, MY-U, MF, MO, TF, Funding acquisition; MY-U, HU, YO, Methodology; HU, MY-U, YO, Project administration; HU, YM, MY-U, YO; Resources; YM, RS, MY-U, Supervision; HU, NI, RS, ET, YO, Writing-original draft; CY, MY-U, Writing- review & editing; CY, MY-U, HU, SY, YO.

## Declaration of competing interest

The authors declare that they have no known competing financial interests or personal relationships that could have appeared to influence the work reported in this paper.

## Appendix A Supplementary data

Supplementary material Image 1
