# Supplementary material for: Coexistence of bone and vascular disturbances in patients with endogenous glucocorticoid excess
Source: Bone Rep. 2022 Aug 11;17:101610. doi: 10.1016/j.bonr.2022.101610 (PMC9398912; doi:10.1016/j.bonr.2022.101610)
Supplement: Supplementary file 1 — Supplementary material [file mmc1.docx]

**Supplementary Table 1. Association between bone and vascular parameters in patients with ACS after adjusting for possible confounders**

| **Association between vertebral fracture and abdominal aortic calcification (n = 77)** | | | |
| --- | --- | --- | --- |
| **Variables** | **OR** | **95% CI** | **p-value** |
| Crude | 3.13 | 1.17-8.94 | 0.026* |
| Model 1 | 3.55 | 1.04-13.3 | 0.048* |
| Model 2 | 3.08 | 0.90-1.13 | 0.077 |
| Model 3 | 3.19 | 0.96-1.15 | 0.063 |

In these analyses, age, gender, BMI, alcohol intake, smoking status, and the presence of diabetes mellitus were adjusted in model 1, and diabetes mellitus of model 1 was replaced by hypertension and hyperlipidemia in models 2 and 3, respectively. Age and BMI were log-transformed. *, p < 0.05 was considered significant.

Abbreviations: ACS, autonomous cortisol secretion; OR, odds ratio; CI, confidence interval; BMI, body mass index.

**Supplementary Table 2. Comparison of clinical characteristics among patients with CS, SCS, and non-functional AT**

| **Variables** | | **CS**  **(n = 17)** | **SCS**  **(n = 80)** | **Non-functional AT (n = 97)** | **p-value ^a^** |
| --- | --- | --- | --- | --- | --- |
| **Baseline parameters** | Age, y | 46.0 [44.0, 59.0] ^b, c^ | 64.5 [47.0, 71.0] | 58.0 [51.0, 67.0] | 0.005* |
|  | Gender, female, % | 64% (11/17) | 61% (49/80) | 53% (52/97) | 0.503 |
|  | Postmenopausal female, % | 36% (4/11) | 63% (31/49) | 73% (38/52) | 0.072 |
|  | BMI, kg/m^2^ | 24.4 [22.7, 26.7] | 23.5 [21.2, 27.0] | 24.1 [21.8, 27.1] | 0.666 |
|  | Alcohol intake, % | 31% (5/16) | 51% (41/79) | 45% (44/97) | 0.300 |
|  | Smoking status, % | 56% (9/16) | 53% (42/79) | 44% (43/97) | 0.429 |
|  | Basal serum cortisol, μg/dL | 16.9 [14.8, 19.2] ^b, c^ | 13.0 [10.2, 18.7] | 12.7 [9.1, 15.9] | 0.003* |
|  | ACTH, pg/mL | 1.0 [1.0, 1.5] ^b, c^ | 8.6 [5.1, 18.2] ^c^ | 24.0 [16.6, 37.1] | <0.001* |
|  | Midnight serum cortisol, μg/dL | 16.2 [15.4, 17.1] ^b, c^ | 5.5 [4.5, 8.1] ^c^ | 2.9 [2.0, 4.2] | <0.001* |
|  | Post-DST cortisol, μg/dL | 16.7 [15.6, 23.0] ^b, c^ | 3.2 [2.3, 6.8] ^c^ | 1.1 [0.8, 1.3] | <0.001* |
|  | 24 h urinary free cortisol, μg/day | 178.0 [160.0, 367.0] ^b, c^ | 50.8 [32.5, 74.6] | 43.7 [31.8, 59.8] | <0.001* |
|  | DHEAS, μg/dL | 20.0 [13.7, 47.0] ^c^ | 40.5 [18.2, 67.7] ^c^ | 92.0 [61.5, 142.5] | <0.001* |
|  | eGFR, ml/min/1.73m^2^ | 85.0 [63.5, 94.5] | 80.4 [71.0, 93.0] | 81.0 [71.0, 93.0] | 0.999 |
|  | Diabetes mellitus, % | 47% (8/17) | 43% (35/80) ^c^ | 22% (22/97) | 0.006* |
|  | Hypertension, % | 82% (14/17) | 69% (55/79) | 52% (50/96) | 0.013* |
|  | Hyperlipidemia, % | 70% (12/17) ^c^ | 53% (43/80) | 36% (35/97) | 0.008* |
| **Bone parameters** | Vertebral fracture, % | 60% (9/15) ^b, c^ | 46% (29/62) | 8% (7/84) | <0.001* |
|  | Severe vertebral fracture, % | 33% (5/15) ^c^ | 12% (8/62) | 2% (2/84) | 0.001* |
|  | BMD at lumbar spine, g/cm^2^ | 0.79 [0.71, 0.89] ^c^  (n = 17) | 0.88 [0.75, 1.02]  (n = 72) | 0.88 [0.80, 1.06]  (n = 87) | 0.034* |
|  | BMD at lumbar spine  Z score | -1.0 [-1.9, -3.0] ^b, c^  (n = 17) | 0.0 [-0.7, 0.7]  (n = 72) | 0.1 [-0.6, 0.9]  (n = 87) | 0.001* |
|  | BMD at femoral neck, g/cm^2^ | 0.55 [0.50, 0.64] ^b, c^  (n = 17) | 0.62 [0.54, 0.72]  (n = 77) | 0.65 [0.60, 0.76]  (n = 87) | 0.002* |
|  | BMD at femoral neck  Z score | -1.8 [-2.3, -1.1] ^b, c^  (n = 17) | -4.0 [-1.1, 0.4]  (n = 77) | -0.3 [-0.8, 0.4]  (n = 87) | <0.001* |
|  | TBS | 1.26 [1.19, 1.35] ^b, c^  (n = 14) | 1.35 [1.30, 1.39]  (n = 68) | 1.37 [1.31, 1.42]  (n = 82) | 0.003* |
|  | Serum calcium, mg/dL | 9.4 [9.1, 9.6] | 9.3 [9.1, 9.5] | 9.3 [9.1, 9.5] | 0.548 |
|  | Intact-PTH, pg/mL | 68.9 [59.5, 89.0] ^b, c^  (n = 15) | 52.3 [36.4, 65.3]  (n = 76) | 43.8 [34.5, 54.7]  (n = 94) | <0.001* |
|  | Urinary calcium-to-creatinine ratio | 0.25 [0.17, 0.28]  (n = 13) ^c^ | 0.16 [0.11, 0.20]  (n = 56) | 0.13 [0.08, 0.20]  (n = 75) | 0.018* |
|  | 25-hydroxyvitamin D, ng/ml | 13.6 [10.1, 21.5]  (n = 7) | 13.9 [9.9, 16.0]  (n = 37) | 14.3 [10.5, 18.5]  (n = 68) | 0.528 |
|  | TRACP-5b, mU/dl | 364 [267, 471]  (n = 14) | 358 [241, 448]  (n = 73) | 323 [251, 433]  (n = 95) | 0.636 |
|  | BAP, μg/L | 15.5 [11.0, 21.5]  (n = 14) | 11.8 [8,7, 14.6]  (n = 72) | 12.5 [9.9, 15.9]  (n = 94) | 0.078 |
| **Vascular parameters** | Abdominal aortic calcification, % | 13% (2/15) | 37% (23/62) ^c^ | 15% (13/84) | 0.006* |
|  | baPWV, cm/s | 1738 [1604, 1895]  (n = 16) | 1657 [1415, 1891]  (n = 80) | 1541 [1365, 1707]  (n = 95) | 0.010* |
|  | Arterial stiffness, % | 43% (7/16) | 36% (29/80) | 21% (20/95) | 0.030* |
| **Coexistence rates of bone and vascular diseases** | Vertebral fracture and arterial stiffness, % | 35% (5/14) ^c^ | 21% (13/62) ^c^ | 2% (2/83) | <0.001* |
|  | Vertebral fracture and abdominal aortic calcification, % | 13% (2/15) | 24% (15/62) ^c^ | 1% (1/84) | <0.001* |

Data are expressed as median [interquartile range] or percentage (number of patients). Severe vertebral fracture was defined as multiple vertebral fractures or a grade 3 vertebral fracture. Arterial stiffness was defined as a baPWV ≥1800 cm/s. a, p-value for the association between the three groups (p < 0.05 was considered significant). b, p < 0.05 different from SCS. c, p < 0.05 different from NFAT.

Abbreviations: CS, overt Cushing’s syndrome; SCS, subclinical Cushing’s syndrome; AT, adrenal tumor; BMI, body mass index; ACTH, adrenocorticotropic hormone; DST, dexamethasone suppression test; DHEAS, dehydroepiandrosterone sulfate; eGFR, estimated glomerular filtration rate; PTH, parathyroid hormone; TRACP-5b, tartrate-resistant acid phosphatase-5b; BAP, bone-alkaline phosphatase; BMD, bone mineral density; TBS, trabecular bone score; baPWV, brachial-ankle pulse wave velocity.

**
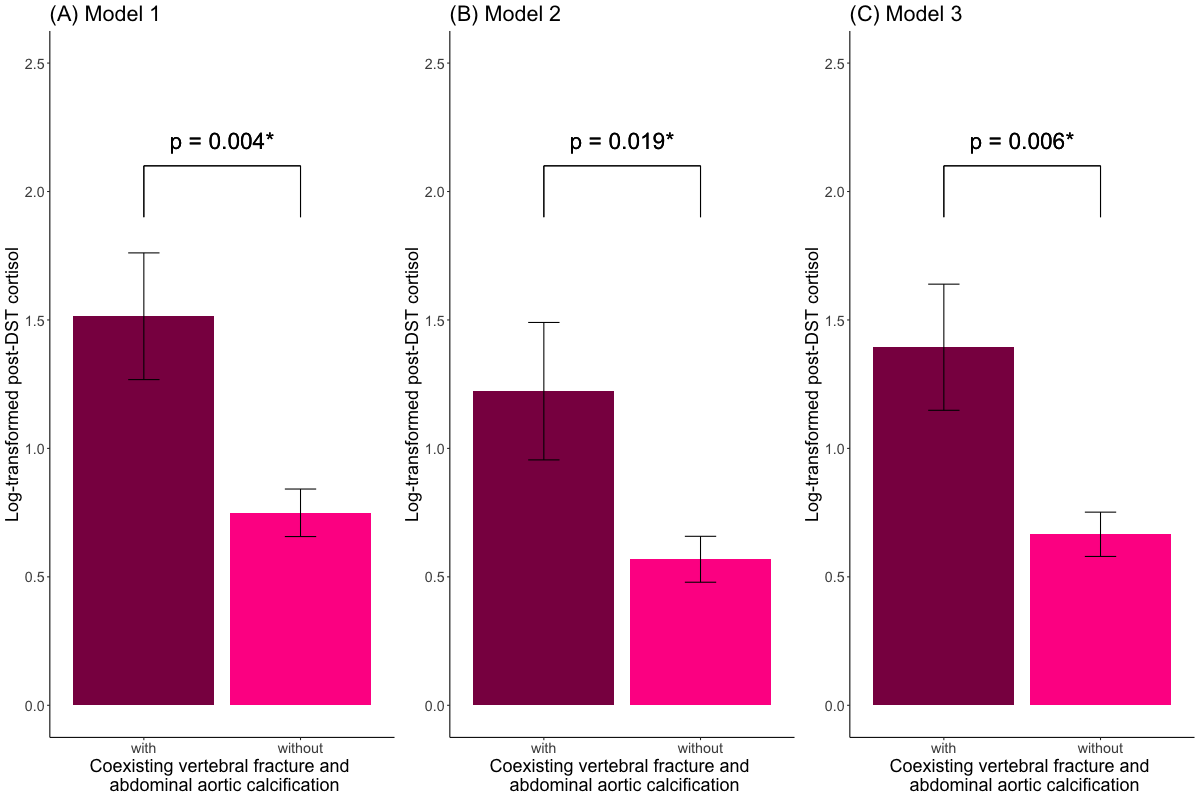
**

**Supplementary Figure 1. Differences in post-DST cortisol between patients with and without coexisting bone and subclinical vascular diseases**

(A) Patients with coexisting vertebral fracture and abdominal aortic calcification (n = 18) had higher levels of post-DST cortisol than those without (n = 143) after adjustment for age, gender, BMI, alcohol intake, smoking status, and the presence of diabetes mellitus in model 1. (B, C) The results were similar when diabetes mellitus in model 1 was replaced by hypertension and hyperlipidemia in models 2 and 3, respectively. In these analyses, data are expressed as the LSM with 95% CI from ANCOVA after adjustment in models 1-3. The post-DST cortisol, age, and BMI were log-transformed. *, p < 0.05 was considered significant.

Abbreviations: CS, overt Cushing’s syndrome; SCS, subclinical Cushing’s syndrome; AT, adrenal tumor; DST, dexamethasone suppression test; ANCOVA, analysis of covariance; LSM, least-square mean; CI, confidence intervals; BMI, body mass index.


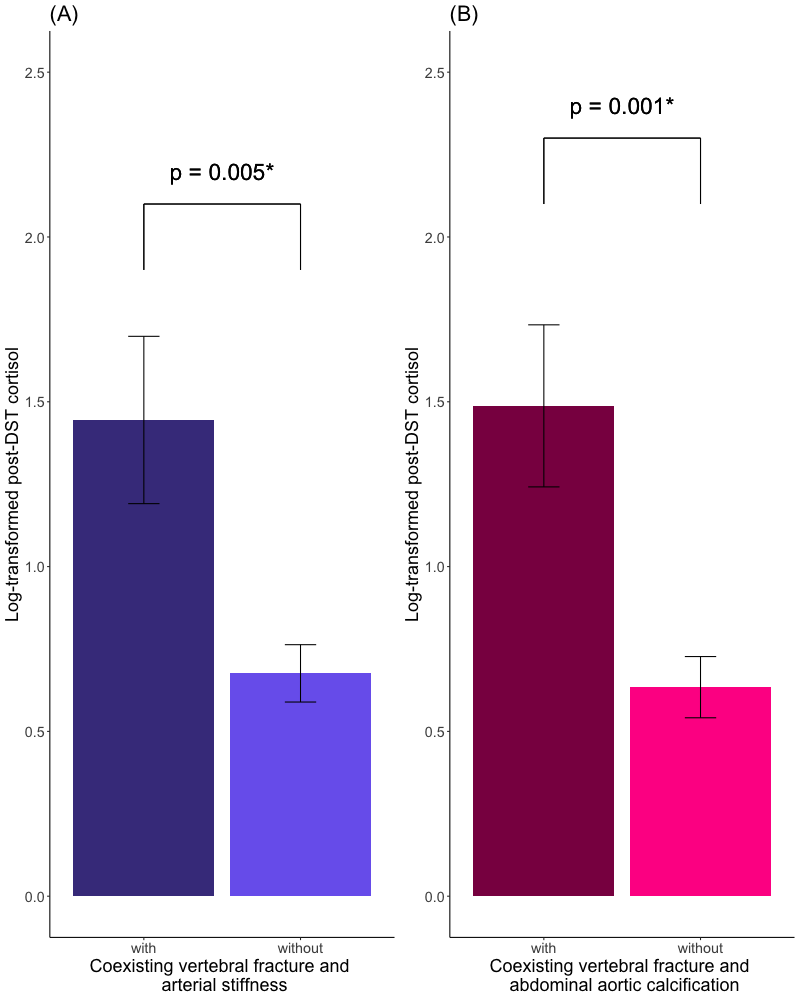


**Supplementary Figure 2. Differences in post-DST cortisol between patients with and without coexisting bone and subclinical vascular diseases (adjusted simultaneously for the 3 variables of the presence of diabetes mellitus, hypertension, and hyperlipidemia)**

(A) Patients with both vertebral fracture and arterial stiffness (n = 20) had higher levels of post-DST cortisol than those without (n = 139) after adjusting for age, gender, BMI, alcohol intake, smoking status, the presence of diabetes mellitus, hypertension, and hyperlipidemia. (B) Similar results were obtained when comparing patients with both vertebral fractures and abdominal aortic calcification (n = 18) with those without (n = 143). In these analyses, data are expressed as the LSM with 95% CI from ANCOVA after adjustment in models 1-3. The post-DST cortisol, age, and BMI were log-transformed. *, p < 0.05 was considered significant.

Abbreviations: CS, overt Cushing’s syndrome; SCS, subclinical Cushing’s syndrome; AT, adrenal tumor; DST, dexamethasone suppression test; ANCOVA, analysis of covariance; LSM, least-square mean; CI, confidence intervals; BMI, body mass index.
